# Patterns of SARS-CoV-2 circulation revealed by a nationwide sewage surveillance programme, the Netherlands, August 2020 to February 2022 

**DOI:** 10.2807/1560-7917.ES.2023.28.25.2200700

**Published:** 2023-06-22

**Authors:** Michiel van Boven, Wouter A Hetebrij, Arno Swart, Erwin Nagelkerke, Rudolf FHJ van der Beek, Sjors Stouten, Rudolf T Hoogeveen, Fuminari Miura, Astrid Kloosterman, Anne-Merel R van der Drift, Anne Welling, Willemijn J Lodder, Ana Maria de Roda Husman

**Affiliations:** 1 Centre for Infectious Disease Control, National Institute for Public Health and the Environment (RIVM), Bilthoven, the Netherlands; 2 Julius Center for Health Sciences and Primary Care, University Medical Center Utrecht, Utrecht University, Utrecht, the Netherlands; 3 Center for Marine Environmental Studies (CMES), Ehime University, Ehime, Japan; 4 Centre for Environmental Safety and Security, National Institute for Public Health and the Environment (RIVM), Bilthoven, the Netherlands; 5 Institute for Risk Assessment Science (IRAS), Utrecht University, Utrecht, the Netherlands

**Keywords:** SARS-CoV-2, qRT-PCR, wastewater-based surveillance, early warning, hospital admissions

## Abstract

**Background:**

Surveillance of SARS-CoV-2 in wastewater offers a near real-time tool to track circulation of SARS-CoV-2 at a local scale. However, individual measurements of SARS-CoV-2 in sewage are noisy, inherently variable and can be left-censored.

**Aim:**

We aimed to infer latent virus loads in a comprehensive sewage surveillance programme that includes all sewage treatment plants (STPs) in the Netherlands and covers 99.6% of the Dutch population.

**Methods:**

We applied a multilevel Bayesian penalised spline model to estimate time- and STP-specific virus loads based on water flow-adjusted SARS-CoV-2 qRT-PCR data for one to four sewage samples per week for each of the more than 300 STPs.

**Results:**

The model captured the epidemic upsurges and downturns in the Netherlands, despite substantial day-to-day variation in the measurements. Estimated STP virus loads varied by more than two orders of magnitude, from ca 10^12^ virus particles per 100,000 persons per day in the epidemic trough in August 2020 to almost 10^15^ per 100,000 in many STPs in January 2022. The timing of epidemics at the local level was slightly shifted between STPs and municipalities, which resulted in less pronounced peaks and troughs at the national level.

**Conclusion:**

Although substantial day-to-day variation is observed in virus load measurements, wastewater-based surveillance of SARS-CoV-2 that is performed at high sampling frequency can track long-term progression of an epidemic at a local scale in near real time.

**Key public health message**

**What did you want to address in this study?**

The primary aim of this study was to estimate SARS-CoV-2 virus loads in sewage in the Netherlands, using data from a comprehensive national surveillance programme. The secondary aim was to compare estimated virus loads in sewage with hospital admission data in order to use the sewage signal as a main indicator of morbidity.

**What have we learnt from this study?**

Virus loads in sewage water can vary by more than two orders of magnitude. The dynamics of the COVID-19 epidemic in wastewater were largely synchronised between municipalities. Estimated virus loads in sewage closely followed the daily number of hospitalisations and case notifications.

**What are the implications of your findings for public health?**

Surveillance of SARS-CoV-2 in wastewater can track in near real-time how the COVID-19 epidemic develops within a defined local area, even in the presence of substantial background signals and day-to-day variation.

## Introduction

The COVID-19 pandemic has posed one of the most severe threats to public health in recent history. Severity and progression of the disease are variable, depending on host risk factors and pathogen variants [1-3]. Transmission can also occur via asymptomatic or pre-symptomatic individuals [4]. These characteristics hamper effective epidemiological surveillance for the infection dynamics, as case notifications are biased, depending on strain-dependent severity of disease, willingness to get tested, testing capacity and public health policies. Epidemiological surveillance based on hospital admissions is less biased but does not provide an accurate picture in the early and late stages of an epidemic when hospitalisations are rare. This is especially true at a local scale (e.g. municipalities) and for variants causing relatively mild disease (such as the Omicron relative to the Delta variant). Serological surveillance for severe acute respiratory syndrome coronavirus 2 (SARS-CoV-2) can provide population-level estimates of the fraction of the population that has been infected [5]. Serological surveys, however, are costly and each survey only provides a cross-sectional snapshot of the population at a single time point.

In contrast with earlier applications of wastewater-based surveillance such as the identification of emerging enteric viruses [6], monitoring of SARS-CoV-2 in sewage can supply quantitative information at a local scale [7]. In the COVID-19 pandemic, the detection of SARS-CoV-2 in faeces has accelerated the introduction of wastewater surveillance in a variety of settings, including airports, hospitals and cities. Wastewater-based surveillance has now been implemented in many countries [8]. However, many of these activities are carried out by local governmental bodies or independent research groups and are often restricted to specific locations. This makes it difficult to compare variations over time and geographically in a broader perspective.

Here we report data and statistical analyses from a national wastewater-based surveillance programme in the Netherlands. Since 7 September 2020, all sewage treatment plants (STPs) in the Netherlands have been providing between one and four samples per week to the Dutch National Institute for Public Health and the Environment (RIVM). Since almost every Dutch household is connected to a sewer, coverage of the programme is close to 100%. Samples are subjected to a standardised extraction protocol and analysed by real-time RT-PCR within 2–4 days after sampling. As measurements of SARS-CoV-2 RNA in sewage are inherently variable and can be left-censored (i.e. not able to detect RNA at low virus loads), our aim was to integrate the available data to provide estimates of the latent true SARS-CoV-2 virus loads over time for all STPs. We analysed data from 1 August 2020 (when 60% of the population had already been included) up to and including 8 February 2022 (when coverage exceeded 99%). Hence, the time series data cover the winter epidemic of 2020/21, the 2021 summer surge after many restrictions had been lifted, and the 2021/22 winter epidemic.

## Methods

### Sampling, RNA extraction, and qRT-PCR

**Figure 1:**
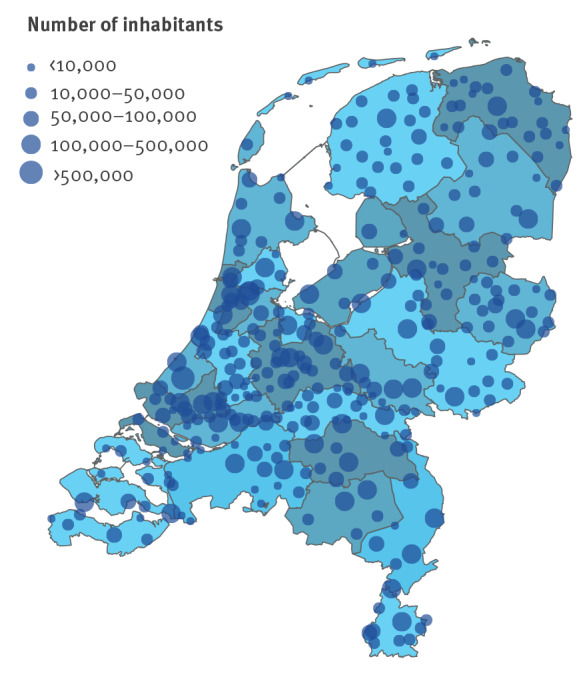
Sewage treatment plants, the Netherlands, 2022 (n = 317)

In the Netherlands, sewage of 99.6% of the population is treated at 317 STPs, of which four closed between start and end of the period of analysis (Figure 1). Coverage of the programme was over 99% of the Dutch population (> 17 million) as only a small fraction of the population in the Netherlands is not connected to a sewage system. The catchment areas of STPs vary in size from covering ca 1,000 to more than 800,000 inhabitants. At each STP, sewage samples were collected 1–4 times per week, and the weekly number of samples generally increased over the study period. Samples contained a volume of 500–1,000 mL, taken from a larger 24 h flow-proportional sample, and were sent to RIVM for analysis. 

Shown are locations of all 317 sewage treatment plants in 2022. Sizes of the dots represent the number of persons serviced by the plant. Background colours represent the 25 safety regions of the Netherlands. Safety regions represent the public bodies whose task is to facilitate regional cooperation in dealing with crises, disasters and disruptions of public order.

At the central laboratory, RNA extraction was performed on each sample using the Viral NA Large Volume kit on the MagNA Pure 96 instrument (Roche, Basel, Switzerland). Detection of SARS-CoV-2 RNA was based on an assay described by the Centers for Disease Control and Prevention (CDC) [9], and qRT-PCR was performed in duplicate for the N1 and N2 targets. In each analysis run we included both positive and negative controls, and added equine arteritis virus (EAV) to the samples to check for uncontrolled viral inhibition. Using an internal standard, quantification cycle (Cq) values were converted to RNA concentrations (RNA/mL). The RNA concentrations of the four measurements were averaged to yield an average RNA concentration. When all four measurements yielded a non-detect result, we reported the sample as negative/left-censored. The RNA/mL values obtained from the RT-qPCR were subsequently divided by the number of permanent residents in the catchment area of the STP and multiplied by 100,000 and by the 24 h water flow of the STP of origin to obtain the number of genome copies per 100,000 inhabitant equivalents. Throughout, we use ‘number of virus particles’ as shorthand for ‘number of genome copies’, and ‘virus load’ as shorthand for number of virus particles per 100,000 normalised by 24 h flow. Finally, municipalities often make use of multiple STPs. Therefore, to transform STP virus loads to virus loads per municipality, the virus loads per STP contributing to a given municipality were weighted by the number of inhabitants serviced by each STP in that municipality.

### Statistical analysis

The basis for the analyses were log_10_-transformed virus concentration data, normalised by 24 h sewage water flow rates (unit: virus particles 100,000 persons/day; henceforth called virus load). The analyses employed a Bayesian multilevel spline model with random effects at the level of the STPs [10,11]. In the model, the expected log_10_-transformed virus load at time *t* and STP *i*, *c_i_(t),* was given by


$$c_{i}\left(t\right)=c\left(t\right)\;+\;d_{i}\left(t\right)$$,

where *c(t)* represents the national trend and the *d_i_(t)* represent the STP-specific deviations. The central goal was to infer the unknown latent virus loads *c_i_(t)* in the presence of measurement error and left-censoring. The national trend *c(t)* was modelled with a penalised spline (p-spline) with cubic b-spline basis functions and 15 equidistant knots (yielding 17 regression coefficients). The results presented here were obtained with a first-order random walk prior for the regression coefficients [11], using an *N(12,1) *prior distribution for the first regression coefficient and an inverse gamma(1, 0.0005) prior distribution for the variance parameter [11]. In this manner, the national log_10_-transformed virus load at the first time point was normally distributed a priori, with a mean of 12 per 100,000 persons per day and approximate 95% prior range of 10–14 per 100,000 persons per day. This provided good coverage for virus loads at the first time points (August 2020). In a similar manner, STP-specific deviations from the trend were modelled with cubic b-splines with 15 equidistant knots (yielding 17 coefficients per STP), and with zero-mean normal prior distributions (*N(0,1)*). Hence, plant-specific deviations were a priori expected to be no more than ca 100-fold (i.e. 2 standard deviations) lower or higher than the trend. Of note, this choice covered almost all (> 99.9%) individual measurements for all STPs over time. 

We also explored a suite of alternatives, e.g. estimating the variance of the deviations, or replacing the p-spline for the national virus load with a b-spline using *N(13,1)* prior distributions for the regression coefficient, and evaluated competing models using the leave-one-out information criterion [12]. Here we report results from the best-fitting model. Throughout, we assume that the log_10_-transformed virus load measurements *Y_it_* at plant *i* and time *t* were normally distributed, *Y_it_~N(c_i_(t), σ)*, and we used an uninformative (improper uniform) prior distribution for *σ>0*.

The probability of virus detection in a sample is determined by the detection limit of the qRT-PCR, the daily flow rates of water through the sewerage, and possibly also by the composition of the sewage. Hence, there is no fixed predefined cut-off for RNA detection that can be applied to all STPs at all time points, and we included in the analyses a two-parameter logistic function that determined the probability of detection. The parameters *c_0_* (load at which detection occurs with a probability of 0.5) and *k* (steepness of the detection curve) were estimated. Hence, denoting the probability of detection by


$$p\left(\text{detection | }c_{i}\left(t\right)\right)=1/\left(1+e^{-k\left(c_{i}\left(t\right)-c_{0}\right)}\right)$$,

a non-detect result added a contribution *1-p(*detection|*c_i_(t))* to the likelihood. In this manner, samples in which no RNA is detected tended to pull the estimated load *c_i_(t)* to lower values. The parameter *c_0_* was given a weakly informative *N(12, 0.5)* prior distribution (a priori of 50% probability of detection ranges from log virus load of ca 11–13), and *k* was provided with an uninformative (improper) uniform prior distribution (*k>2*). The event of a detection was Bernoulli-distributed, *D*~Bernoulli*(p(*detection|*c_i_(t)))*. Putting it together, the log-likelihood contributions were given by


$$L_{it}=\left\{\begin{array}{cc} f_{D}\left(1;p\left(\text{detection}\right)\right)+f_{Y}\left(Y_{it};c_{i}\left(t\right),\sigma \right) & \text{if RNA was detected at STP}\text{ i}\text{ and time }\text{t}\;\\ f_{D}\left(0;p\left(\text{detection}\right)\right) & \text{if RNA was not detected, } \end{array}\right.$$

where *f_D_* and *f_Y_* denote the log Bernoulli and log normal probability densities, respectively.

To tie the results at the STP level to other organisational levels (municipality, safety region, national) we applied posterior weighting of the estimated virus loads (i.e. the exponentiated estimated log loads), where weighting was proportional to the numbers of persons contributing to the various organisational units. Demographic data were obtained from Statistics the Netherlands using the census of 1 January 2020 [13], with the exception of the municipal mergers of January 2021 and 2022 which we added manually to the demographic data. Specifically, the expected log_10_-transformed virus load at time *t *in municipality *i*, *b_i_(t)*, was given by


bit = log10⁡∑jIi,j × 10cj(t)∑jIi,j,

where we summed over all STPs *j*, and *I_i,j_* were the number of inhabitants in municipality *i* serviced by STP *j*. Log-transformed virus loads at other organizational levels (safety region, province) were calculated similarly. Of note, the analyses reported here included all but the three overseas extraordinary municipalities (Bonaire, St Eustatius and Saba).

We analysed sewage data of more than 47,000 measurements spanning the period from 1 August 2020 (when the majority of STPs had been included in the national programme) up to and including 8 February 2022. For comparison of the results with other epidemic indicators, we also made use of the daily incidence of hospital admissions (cases per 100,000 persons) and the daily number of positive tests, both stratified by municipality. All analyses were performed with Stan (version 2.21.0) and R (version 4.1.3), using RStan (version 2.21.2) as interface to Stan [14]. We ran 10 Markov chain Monte Carlo chains in parallel and based the analyses on 1,000 samples from well-mixed chains, where we applied 1/4 thinning to minimise correlations between samples. Data, scripts and figures are available in the online repository at https://github.com/rivm-syso/SARS-CoV-2_sewage.

## Results

### Descriptive statistics

The Dutch sewage surveillance programme for SARS-CoV-2 was initiated in the first months of 2020. Since its inception, the number of included STPs has increased over time such that 80 STPs provided samples in August 2020, and all 313–317 STPs provided samples from September 2020 onwards. An overview of the location and size of STPs in the Netherlands is provided in Figure 1 and shows that STPs in the densely populated western parts of the Netherlands are generally substantially larger than those in other regions. Approximately 3% of all measurements (1,479 of 46,448) yielded a non-detection, and those were concentrated in the early stages of the epidemic when virus loads were still low (< 10^12.5^). It is noteworthy that STPs often process sewage from multiple municipalities, and it is not uncommon that a municipality is served by multiple STPs. Hence, STPs do not follow the hierarchical organisational structure of the Dutch administrative divisions, which makes averaging from STP-level analyses to municipalities, provinces and safety regions not straightforward.

### Estimation of viral loads

**Figure 2:**
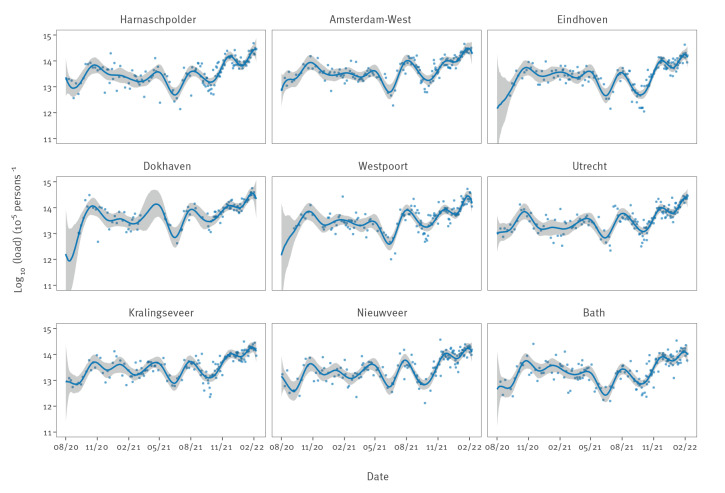
SARS-CoV-2 loads in sewage and fit of the model for the largest sewage treatment plants, the Netherlands, August 2020–February 2022 (n = 1,319 data points)

Figure 2 shows the latent virus loads for the nine largest STPs. Results for all 317 STPs are available in the online repository at https://github.com/rivm-syso/SARS-CoV-2_sewage. In general, the model described the data well, and indicators suggested that the estimation procedure yielded satisfying results. Specifically, the scale reduction factor was close to 1, there were no divergent transitions, components of the posterior distribution were located well within the support of the prior distributions yielding strong posterior contraction, there were no systematic deviations of residuals from posterior medians. Further, in Supplementary Figure S1 we show that approximately 95% of observations were within the 95% posterior prediction intervals.

SARS-CoV-2: severe acute respiratory syndrome coronavirus 2.

The total number of persons connected to these plants was 3,641,251 [13], representing 21.0% of the population covered by all 317 STPs in the Netherlands. Dots represent virus load measurements; lines show the posterior medians of the fitted virus loads with 95% credible intervals (grey bands). Dates on the x-axis are given in numeric format, i.e. month/year. Figures and underlying data for all STPs are available at https://github.com/rivm-syso/SARS-CoV-2_sewage. STPs are sorted by size (top left to bottom right).

**Figure 3:**
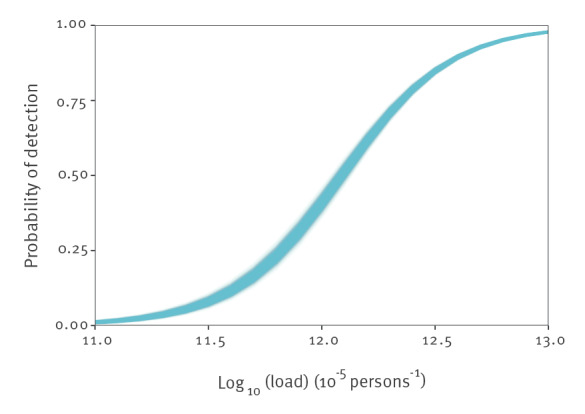
Estimated probability of SARS-CoV-2 RNA detection in sewage samples as a function of the log_10_-transformed sewage load, the Netherlands, August 2020–February 2022 (n = 1,000 samples from the posterior distribution)

Estimated virus loads were generally low in August and September 2020 and were estimated between 10^12^–10^13^ virus particles per 100,000 inhabitants per day. At these virus loads, a sizeable fraction of samples can yield a non-detection, especially at the lower end of this range. This is illustrated in Figure 3, which shows the probability of detection as a function of virus load, estimated from the joint data of all STPs. The estimated probability of detection was close to 0 at a virus load of 10^11^, close to 50% at a virus load of 10^12^, and almost 1 at a virus load of 10^13^. Estimated parameters of the detection function were *c_0 _* = 12.1 (95% credible interval (CrI): 12.1–12.2) for the posterior median of the logistic midpoint, and *k *= 4.2 (95% CrI: 4.0–4.4) for the posterior median of the logistic growth rate.

SARS-CoV-2: severe acute respiratory syndrome coronavirus 2.

Between August 2020 and November 2020, virus loads increased by more than one order of magnitude to a peak value close to ca 10^14^ particles per 100,000 per day in many STPs. Thereafter, virus loads started to decline gradually in all STPs. From May 2021 onwards, when the vaccination coverage was strongly increasing, virus loads started to decrease at a faster pace until the early summer. Subsequently, virus loads increased and reached a peak in early August 2021 by the temporary lifting of restrictions for vaccinated persons. Finally, from October 2021, virus loads started increasing again, first to just under 10^14^ particles per 100,000 per day in most STPs in December 2021, and subsequently to well over 10^14^ and even up to 10^15^ particles per 100,000 per day in February 2022.

Overall, we observed that trends in virus loads corresponded to main epidemiological events. This was true even though we estimate that measurement variation was substantial. In fact, the posterior median of the standard deviation of the observation model was *σ*=0.353 (95% CrI: 0.351–0.355), such that individual measurements could be 10^2σ ^≈ 5-fold lower or higher than the estimated virus load. This, however, is still substantially smaller than the 100- to 1,000-fold increases and decreases in virus loads from epidemic troughs to peaks and vice versa.

### Relation to hospital admissions

**Figure 4:**
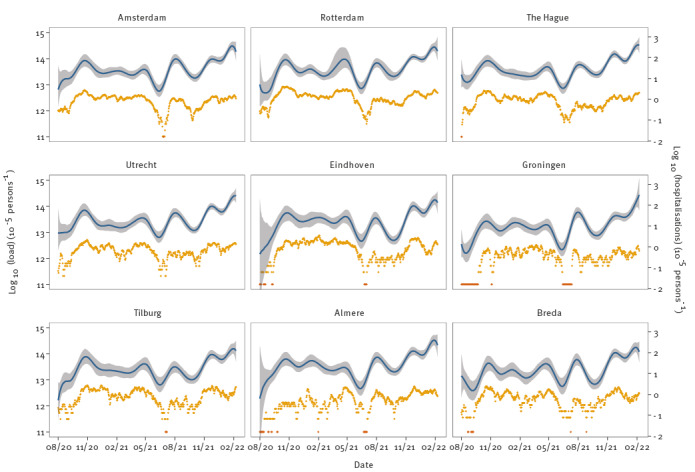
Estimated log-transformed SARS-CoV-2 loads for the nine largest municipalities in the Netherlands with log-transformed number of hospitalisations, the Netherlands, August 2020–February 2022 (n = 19,146 hospital admissions)

The estimated virus loads in the nine largest municipalities in the Netherlands are presented in Figure 4. We obtained these municipal estimates from the STP estimates by weighting the relevant posterior virus loads by the number of inhabitants in the focal municipality. The patterns were similar to those in Figure 2 for the STPs. The peaks and troughs at the municipality levels matched the estimated peaks and troughs at the STP level. Estimated virus loads for all 345 municipalities are available in the online repository and were broadly similar to the results presented in Figures 2 and 4. For comparison with other epidemic indicators, Figure 4 also shows the log-transformed daily incidence of hospital admissions. In addition, we provide in Supplementary Figure S2 the estimated virus loads in the nine largest municipalities with the daily number of positive tests, showing a similar correspondence between virus loads and case notifications as we observed between virus loads and hospitalisations.

SARS-CoV-2: severe acute respiratory syndrome coronavirus 2.

Yellow dots: virus load measurements; lines: posterior median of the STP-weighted national virus load with associated 95% credible band (shaded). Hospitalisations are retrieved from the Dutch open data [31]. Hospitalisations are shown as a 7-day centred moving average to smooth out day-of-week effects, and zero incidences are plotted in red. Notable events in the period October 2020 to February 2022 include a vaccination campaign starting in January 2021 and a campaign for booster vaccination starting in November 2021. Furthermore, the SARS-CoV-2 Alpha, Delta and Omicron variants were successively dominant in the Netherlands and responsible for most infections from February 2021, June 2021 and December 2021, respectively. Hospitalisations in Amsterdam include those from Weesp as those municipalities were merged in March 2022 and the open data we used had been retroactively modified as a result. The total number of persons in these nine municipalities was 3,508,948, representing 20.2% of the population [13]. Dates on the x-axis are given in numeric format, i.e. month/year.

**Figure 5:**
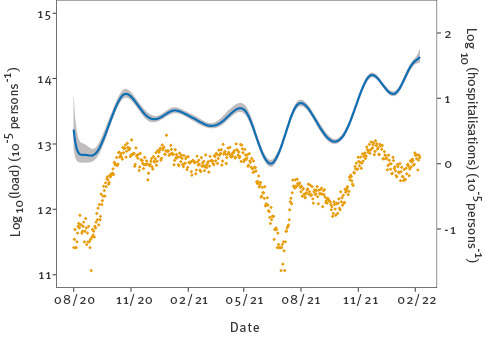
Estimated national log-transformed SARS-CoV-2 loads in sewage with national log-transformed daily number of hospitalisations, the Netherlands, August 2020–February 2022 (n = 82,103 hospital admissions)

Finally, Figure 5 shows the inferred log-transformed virus loads in the Netherlands, using the population-weighted STP estimates, together with the log-transformed daily total number of hospitalisations. Again, overall patterns of estimated virus loads were similar to those shown in Figures 2 and 4. Interestingly, epidemic troughs and peaks in the national estimate were less pronounced than those observed in most STPs. For instance, virus load estimates were as low as 10^12^ particles per 100,000 per day in many STPs in August 2020 but almost 10^13^ at the national level, and almost 10^15^ in the February 2022 peak but only just over 10^14^ at the national level. The fact that local epidemics were not fully synchronised is the reason that the national trend was less pronounced than those in the individual STPs (Figure 2). In fact, there was up to 10-fold variation from one STP to the next in peak height and trough depth, and up to 4 week shifts in the timing of the peaks and troughs.

SARS-CoV-2: severe acute respiratory syndrome coronavirus 2.

The blue line shows the posterior median of the STP-weighted national virus load with associated 95% credible band (shaded), and the yellow dots represent the daily number of hospital admissions [31]. Dates on the x-axis are given in numeric format, i.e. month/year.

## Discussion

More than 99% of the Dutch population is connected to the sewerage and thus to an STP. This makes it possible to perform high resolution and near real-time surveillance of pathogens that are shed in faeces, urine and other excreta and secreta [15,16]. However, sewage is a complex matrix to analyse [17], and proper controls are necessary to accurately quantify SARS-CoV-2 RNA. It is known that the amount of precipitation and the composition of industrial wastewater that is mixed in with the sewage of the general population will affect SARS-CoV-2 RNA concentrations determined by RT-PCR [18]. Still other factors that can affect measurements include the length of sewer lines and ambient temperature [19]. Here we have shown that even when there is substantial noise or day-to-day variation in RNA concentrations detected in sewage, it is possible to reliably estimate variations in underlying latent SARS-CoV-2 virus loads over time. This is possible because virus loads in sewage increase and decline exponentially during epidemics, often by more than two orders of magnitude. These very strong variations over time dwarf the noise in the signal, which is determined by the standard deviation of the log_10_-transformed virus load measurements (*σ).* In the analyses, the standard deviation is estimated at 0.353, such that the estimated latent virus load can be approximately fivefold lower or higher than the individual measurements.

Our analyses using multilevel splines provide a convenient research tool to analyse sewage data, as it enables a natural borrowing of information from the national virus load and samples taken around the same time. This has not hitherto been included in other smoothing approaches of sewage data [20]. In this manner, we have been able to infer trends over time in the latent true virus loads for all 317 STPs in the Netherlands, as well as for a variety of other organisational levels (e.g. municipality, safety region, province and national). Specifically, we provided estimates of the latent virus loads at all time points and in particular for STPs on days when no measurements are available. In principle, our method of analysis can be extended further to any level of organisation. In addition, subsampling from sewer lines in STPs with an unusually high virus load is also possible [21]. Especially in large STPs (such as Amsterdam-West and other STPs in Figure 2) there may be substantial differences between city districts with respect to SARS-CoV-2 prevalence, possibly associated with risk factors such as vaccination coverage and socioeconomic status [22].

Although our analyses provide an adequate fit to the data, we do not claim that the results are optimal for all SPTs at all time points. Rather, they provide a parameter-sparse description of the data, linking the 317 STP time series through the national virus load. This seemed to work well in general, but it should be noted that the model has difficulty following very sudden changes in virus load data. A prominent example were the sudden and strong drops in virus loads around October 2021. This is only partially resolved by adding more knots to the splines, at the cost of greatly increasing computation times (data not shown). Future extensions could also focus on adding STP-specific measurement noise or perhaps even directly modelling the PCR data and water flow rates jointly.

Our statistical modelling has provided a description of trends in virus loads at a local level by weighing all available wastewater measurements. In contrast, other modelling studies have focused on relating national or subnational virus loads to case notification data or hospitalisations using mechanistic modelling, with the aim to estimate the incidence and prevalence of the number of infections over time ([23-25] and references therein). The two approaches serve different purposes, and each has its strengths and limitations. A main strength of the mechanistic modelling is that all parameters have a biological interpretation, and that these analyses can be used for scenario studies. The results from such analyses, however, depend critically on model assumptions and are surrounded with large uncertainties. Our analyses do not yield a mechanistic interpretation but give precise estimates of latent virus loads that arguably are less dependent on specific model assumptions.

Our analyses provide steps to integrate noisy individual virus load measurements in sewage into more smooth estimates of the underlying latent virus load. This is valuable in itself, but we believe that the future value of the analyses will be mostly in combining the sewage data with other epidemic indicators such as hospitalisations or positive tests [26,27]. In addition, since the infrastructure of receiving sewage samples is in place, the detection of other viruses can and will be added to the Dutch sewage surveillance programme. These include rotavirus and enteroviruses but also respiratory syncytial virus and influenza viruses [28,29], thus providing a comprehensive and general surveillance tool to support pandemic preparedness. Moreover, sewage surveillance has also shown potential as a means to track antimicrobial resistance in the population [30]. In principle, our methods of analysis can be directly applied to other targets and can deal with noise and unbalanced data in a systematic manner.

## Conclusion

Wastewater-based surveillance of SARS-CoV-2 can track long-term epidemic progression at a local scale in near real time, even in the presence of substantial noise and day-to-day variation. This can support public health authorities by early notification of local increases in virus circulation in a semi-endemic situation with decreasing numbers of case notifications and hospital admissions.

## Supplementary Data

1055000 Supplement
