# Supplementary material for: Patterns of SARS-CoV-2 circulation revealed by a nationwide sewage surveillance programme, the Netherlands, August 2020 to February 2022
Source: Euro Surveill. 2023 Jun 22;28(25):2200700. doi: 10.2807/1560-7917.ES.2023.28.25.2200700 (PMC10288829; doi:10.2807/1560-7917.ES.2023.28.25.2200700)
Supplement: Supplement [file 22-0700_vanBOVEN_supplement.pdf]

This supplementary material is hosted by *Eurosurveillance* as supporting information alongside the article Patterns of SARS-CoV-2 circulation in the Netherlands, August 2020-February 2022, revealed by a nationwide sewage surveillance program, on behalf of the authors, who remain responsible for the accuracy and appropriateness of the content. The same standards for ethics, copyright, attributions and permissions as for the article apply. Supplements are not edited by *Eurosurveillance* and the journal is not responsible for the maintenance of any links or email addresses provided therein.

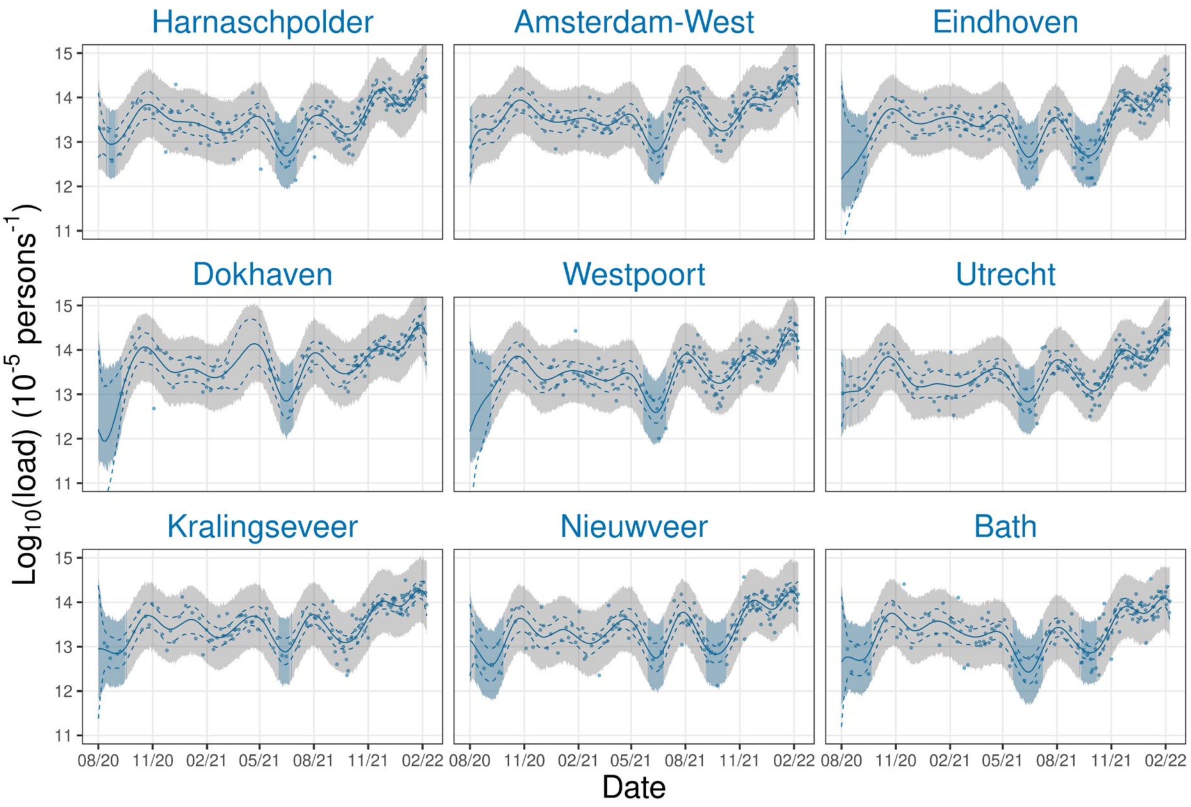

Figure S1. SARS-CoV-2 loads in sewage (dots) and fit of the model (solid lines) for the nine largest STPs in the Netherlands. STPs are sorted by size (top left to bottom right). Total number of persons connected to these plants is 3,641,251 (Statistics Netherlands, 2020), representing 21.0% of the population covered by all 317 STPs in The Netherlands. Lines show the posterior medians of the fitted virus loads with limits of the 95% credible intervals (dotted lines). Grey bands represent the 95% posterior prediction intervals, and illustrate that most measurements lie within the prediction interval. More precisely and looking at averages for all STPs, we find that of 47,927 measurements there were 46,448 in which RNA was detected (i.e. which did not result in a non-detect). Of these, 44,451 were inside the 95% posterior prediction interval. Furthermore, for 1,202 of the 1,479 measurements which resulted in a non-detect the posterior prediction interval consisted of more than 5% non-detects. In other words, approximately (44,451 + 1,202)/47,927 ~ 95.3% of all measurements were within the 95% posterior credible interval. Of note, coverages are calculated by conditioning on samples in which RNA was detected (i.e. excluding non-detects). As a consequence there is a small overrepresentation of samples with relatively high viral counts (relative to the estimates latent viral load) in periods with high fraction of non-detects. These are indicated in the figure below by blue bands (≥2.5% non-detects).

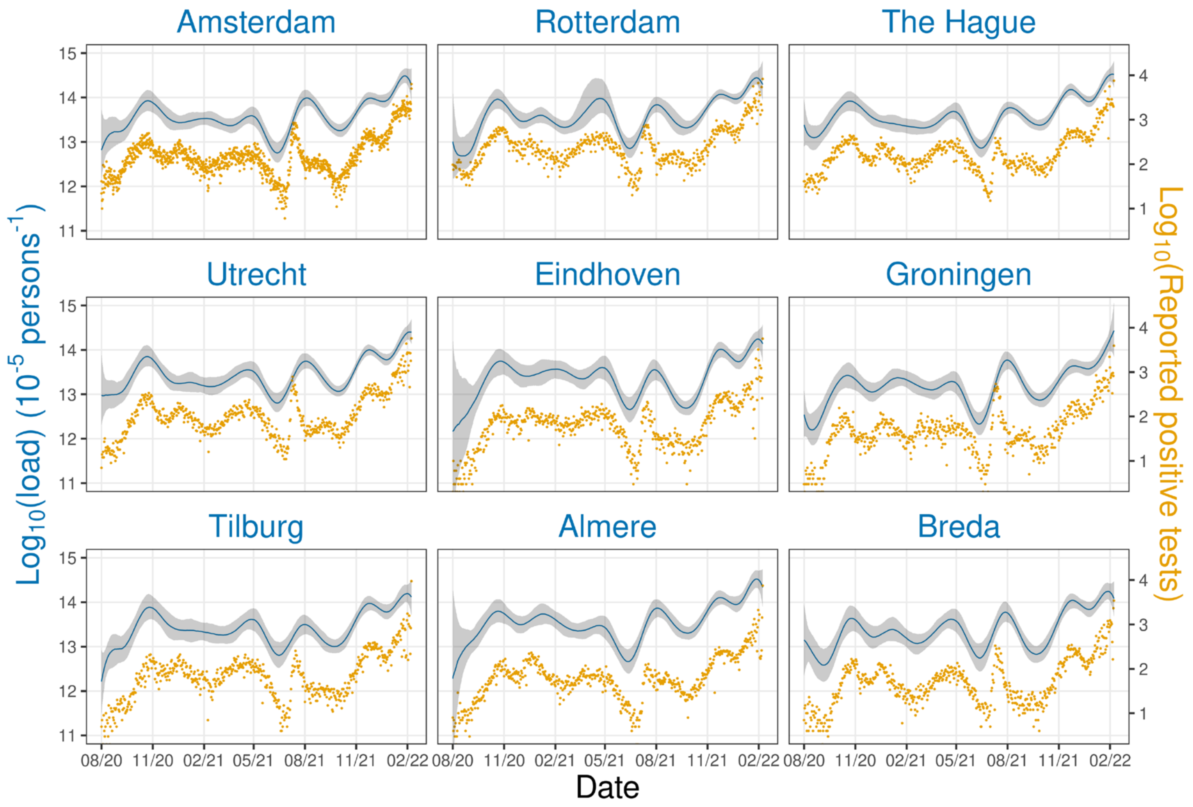

Figure S2. Estimated log-transformed SARS-CoV-2 loads for the nine largest municipalities in the Netherlands (lines) with log-transformed number of case notifications (dots). The line shows the posterior median of the STP-weighted national virus load with associated 95% credible band (shaded). Case notifications are retrieved from the Dutch open data (https://data.rivm.nl/covid-19/). Case notifications in Amsterdam include those from Weesp as those municipalities were merged in March 2022 and the open data we use has been retroactively modified as a result. Total number of persons in these nine municipalities is 3,508,948, representing 20.2% of the population (January 2020 census). Data are available at https://github.com/rivm-syso/SARS-CoV-2_sewage.
